# Midterm results of mitral valve repair with lifting annuloplasty strip for acute phase infective endocarditis

**DOI:** 10.1186/s13019-015-0368-9

**Published:** 2015-10-31

**Authors:** Sung Jun Lee, Hyun Suk Yang, Jun Seok Kim, Je Kyoun Shin, Jae Sung Son, Meong Gun Song, Hyun Keun Chee

**Affiliations:** 1 Department of Thoracic and Cardiovascular Surgery, Konkuk University Medical Center, Research Institute of Medical Science, Konkuk University School of Medicine, Seoul, South Korea; 2 Department of Cardiovascular medicine, Konkuk University Medical Center, Research Institute of Medical Science, Konkuk University School of Medicine, Seoul, South Korea; 3 Department of Pediatric Cardiology, Konkuk University Medical Center, Research Institute of Medical Science, Konkuk University School of Medicine, Seoul, South Korea

**Keywords:** Endocarditis, Mitral Valve Insufficiency, Repair Material

## Abstract

**Background:**

Mitral valve repair is favored over replacement due to superior outcomes. However, extensive infective endocarditis (IE) often has been considered unreconstructable. We retrospectively analyzed the mid-term outcomes of an individualized repair approach using a lifting annuloplasty strip.

**Methods:**

Between December 2007 and March 2014, 27 consecutive patients with acute single mitral valve IE (age 43 ± 16 years, 11 men) underwent lifting mitral annuloplasty (LMA) with a strip (Mitracon® strip, 28 mm in 4, 32 mm in 10, and 34 mm in 13). Blood culture was positive in 70 % (*Streptococcus* 10, *Staphylococcus* 4, HACEK 3, *Enterococcus* 1, Gram negative bacilli 1). One case (4 %) had a previously repaired mitral valve—the repair was redone. Via right thoracotomy (74 %) or median sternotomy (26 %), repair was performed by removal of vegetation and resection of infected tissue, the defect typically then being repaired using a bovine pericardial patch (81 %). Artificial chordae were formed in 5 patients. Nine (33 %) of them had posterior leaflet augmentation (PLA) to get sufficient coaptation height. Clinical and echocardiographic follow-up were performed.

**Results:**

Compared with preoperative ones, postoperative echocardiograms revealed decreases of left ventricular (LV) end-diastolic dimensions (57.2 ± 6.3 versus, 45.4 ± 6.2, or 44.8 ± 4.1 mm, all *p* < 0.01). The LV ejection fraction decreased immediately, but recovered (64.4 ± 9.6 % vs. 54.5 ± 9.8 %, or 65.2 ± 6.1 %, *p* = 0.002, *p* = 1.000, respectively). The latest follow-up echocardiograms (median 28 months) universally showed no or minimal regurgitation, with a preserved mitral valve opening area (2.27 ± 0.48 cm^2^). During the clinical follow-up (median, 54 months), one (3.7 %) death was observed (in-hospital, due to biliary sepsis and pneumonia). There was no reoperation or major cardiovascular event. The 5 year survival rate was 96.3 %.

**Conclusions:**

The repair technique of LMA and/or PLA in patients with IE achieved good structural and functional outcomes as well as an excellent 5 year survival rate. An individualized repair approach should be recommended in patients with acute phase IE.

## Background

Despite advanced diagnostic modalities and therapeutic techniques, infective endocarditis (IE) remains a dangerous condition with an incidence ranging from 16 to 62 cases per million person-years [1]. Treatment for IE consists of antibiotic therapy or surgery, or both. Most patients receive a total of 6 weeks of antibiotics therapy regardless of the timing of surgery [2]. Indications for surgical treatment in patients with IE include congestive heart failure, periannular extension of IE, systemic embolism, cerebrovascular complications, persistent sepsis, and large vegetations with a risk of embolization [3].

In the setting of degenerative mitral valve regurgitation, mitral valve repair is preferred over mitral valve replacement, but for treatment of mitral regurgitation due to IE, mitral valve replacement is preferred because of concerns about the durability of mitral valve repair, recurrence of IE, and mitral regurgitation [4–7]. But recent published clinical trials have suggested that mitral valve repair may be feasible and offer better survival compared with mitral valve replacement even in patients undergoing surgery for IE [1, 8, 9].

The aim of the present single-center, consecutive patient study is to investigate the outcomes of mitral valve repair using lifting mitral annuloplasty (LMA) during acute phase IE having extensive destruction of the mitral valve.

## Methods

### Study population

The Konkuk University Medical Center Institutional Review Board approved this retrospective study and waived the need for individual patient consent. The clinical records of patients who underwent mitral valve surgery for IE were reviewed from December 2007 to March 2014. A total 39 mitral valve open heart surgeries were performed during the study period for correction of acute phase IE in the mitral valve. Among them, there were 29 single mitral valve surgeries, the rest double valve, including the aortic valve. Of the 29 cases, two patients underwent mitral valve replacement—one underwent prior open heart surgery for mitral valve replacement and MAZE for rheumatic mitral valve stenosis and atrial fibrillation, the other underwent mitral valve repair for unknown etiology elsewhere. The study cohort of single mitral valve repairs due to acute phase IE therefore comprised 27 consecutive patients. The mean age was 43 ± 16 years (range, 21–81), the baseline characteristics are summarized in Table 1.

**Table 1:** Preoperative characteristics of the 27 patients who underwent lifting mitral annuloplasty

Variables	No. (%)
Age (years)	43 ± 16
Male	11 (41)
Indication for surgery (≥ moderate to severe MR) and,	
Systemic embolism with large vegetations	12 (44)
Heart failure with acute pulmonary edema	7 (26)
Septic shock	3 (11)
Persistent fever and positive blood cultures > 7 to 10 days	1 (4)
LV EF (%)	64.4 ± 9.6
MR ERO (cm^2^)	0.59 ± 0.30
Positive blood culture	19 (70)
*Streptococcus*	11
*Mitis*	4
*Sanguinis*	2
*Viridans*	2
*Pneumoniae*	1
*Gordonii*	1
*Enterococcus fecalis*	1
*Staphylococcus*	4
MRSA	3
MSSA	1
HACEK group	3
Gram negative bacilli	1
Valvular pathology	
Native mitral valve	26 (96)
Myxomatous, prolapse	10
Ruptured chordae	9
Rheumatic	4
Unclear, destructed by vegetations	3
Status post mitral valve repair, elsewhere	1 (4)

*MR* Mitral regurgitation, *LV EF* Left ventricular ejection fraction, *ERO* Effective regurgitant orifice, *MRSA* Methicillin- resistant *Staphylococcus aureus*, *MSSA* Methicillin-sensitive *Staphylococcus aureus*, *HACEK Haemophilus, Aggregatibacter, Cardiobacterium, Eikenella*, and *Kingella*

Acute phase endocarditis was clinically diagnosed by the modified Duke criteria [10], and surgically confirmed in the entire study population. Nineteen patients (70 %) were blood culture-positive IE. The most common microorganisms were *Streptococcus* in 11 patients (58 %)—*Streptococcus mitis* in 4, *Streptococcus sanguinis* in 2, Other *Viridans streptococci* in 2, Streptococcus pneumoniae in 1, *Streptococcus gordonii* in 1, and *Enterococcus faecalis* in 1. Next were *Staphylococcus* in 4 (21 %) (Methicillin-resistant *Staphylococcus aureus* [MRSA] in 3, and Methicillin-sensitive *Staphylococcus aureus* in 1), the *Haemophilus parainfluenza* in 3 (16 %), and other gram-negative bacilli in 1 (5 %). Culture-negative endocarditis was defined as endocarditis in which no microorganism could be identified either on serial blood culture or in cultures made from the explanted valvular tissue of patients presenting with the clinical picture of endocarditis. Its presentation was significant with systemic embolism in 12 patients (44 %): embolic stroke was the first presentation in 9 patients, and there were other combined systemic embolic infarctions in the spleen (2 patients), kidney (one patient), or other peripheral extremities (2 patients). The presentation with acute heart failure with acute pulmonary edema was 26 % and with septic shock, 11 %. The preoperative chest X-ray revealed pulmonary edema in 7 patients while electrocardiograms showed atrial fibrillation in 3 patients (paroxysmal atrial fibrillation in 1).

The preoperative echocardiogram showed a mean left ventricular (LV) ejection fraction (EF) of 64.4 ± 9.6 % (range, 35–79 %). One patient had prior mitral valve ring annuloplasty elsewhere, and the others showed native mitral valves with leaflet prolapse or chordae rupture, and suspicious underlying rheumatic pathologies (4 patients). The mitral vegetations were extensive in both anterior and posterior leaflets in 15 patients, mainly anterior in 9 patients, and posterior in 3 patients. The amount of mitral valve regurgitation was moderate or severe with an effective regurgitant orifice (ERO) of 0.59 ± 0.30 cm^2^ by the proximal isovelocity surface area method [11].

### Timing of surgery

The timing for surgery varied. The median period from admission to operation was 4 days (interquartile range, 1– 9). As a referral center, the actual interval from diagnosis to operation was 20.9 ± 14.0 days, and according to the culture-driven antimicrobial therapy [12] preoperative intravenous antibiotics were prescribed for 21.0 ± 13.6 days.

### Operative procedure

Right anterolateral thoracotomy, as a standard approach of surgery for mitral valve regurgitation in our center, was performed in 74 % (20/27) of patients. Under general anesthesia with double-lumen intubation, patients were positioned in left decubitus. Cardiopulmonary bypass was initiated using peripheral cannulation through the right internal jugular vein, right femoral vein, and right femoral artery, under transesophageal echocardiography guidance. Median sternotomy was performed in 26 % (7/27) of patients who had obstructive or restrictive lung disease on the pulmonary function test (such as measuring forced expiratory volume in less than 1 s) or who underwent cardiac surgery via right thoracotomy previously. All operations were performed using a standard cardiopulmonary bypass with crystalloid cardioplegia and mild hypothermia. Operative data are summarized in Table 2.

**Table 2:** Operative data

Variables	No. (%)
Approach	
Right thoracotomy	20 (74)
Left thoracotomy	7 (26)
Repair techniques	
Lifting annuloplasty with a strip	27 (100)
Leaflet patching repair	22 (81)
Posterior leaflet augmentation	9 (33)
Artificial chordae formation	5 (19)
Lifting annuloplasty strip size	
28 mm	4 (15)
32 mm	10 (37)
34 mm	13 (48)
Concomitant procedure	4 (15)
Cox-Maze procedure	2
Tricuspid annuloplasty	1
Direct closure of persistent foramen ovale	1

All mitral valve repairs were performed through the left atrium via the groove of Sondergaard. All the visible vegetations and infected tissues were eliminated. In 22 patients (81 %), including the 3 patients with a previous perforation from destructive IE, the ruptured hole in mitral valve leaflets formed by the removal of vegetation or slicing of the leaflet was repaired with bovine pericardium (Supple Peri-guard® bovine pericardium repair patch, 6 cm × 8 cm, Synovis® Surgical, Jisang International, Inc.). Chordal replacement with expanded polytetrafluoroethylene sutures was performed in 5 (19 %) patients, posterior leaflet augmentation (PLA) in 9 (33 %) [13]. As the final step, in all patients, LMA was performed using a mitral strip (Mitracon®, ScienCity Co., Seoul, Korea), flat and flexible polyethylene terephthalate, 5 mm wide with two thick margins and a thin gully between them [14]. The LMA technique was described in in detail previously [14]. Interrupted mattress sutures of non-absorbable multifilament material are placed in the posterior mitral annulus, beginning at the anterolateral commissure to posteromedial commissure. Six sutures are generally required. The difference from typical annular sutures is the placement: in LMA, annular sutures pass through the left atrial (LA) wall above the posterior mitral annulus, going slightly through the annulus and then out through LA wall. This type of suture has the effect of lifting the posterior annulus to the LA side. Since 2007, our institution has performed this type of annular suture. In 9 (33 %) patients, to achieve a sufficient coaptation length (≥5 mm) to prevent development of mitral regurgitation, PLA was performed using an elliptical pericardial patch [15]. Other concomitant cardiac procedures were performed in 4 patients (15 %), including the maze procedure, tricuspid annuloplasty, and the direct closure of patent foramen ovale.

### Post-operative antibiotic therapy

In patients with a positive operative tissue culture (two cases, MRSA, *Enterococcus faecalis*), full coverage of antimicrobial therapy was given, starting from the time of surgery; however, in most patients with a negative operative tissue culture, the duration of antimicrobial therapy was calculated from the preoperative antibiotics for 4 to 6 weeks, and minimum one week of post-operative period even if the duration extended the overall course. The duration of postoperative antibiotic therapy was 22.4 ± 11.1 days.

### Statistical analysis

Statistical analysis was performed with dBSTAT (DBSTAT Version 5. Chuncheon, Korea: DBSTAT Co.; 2010. http://dbstat.com). Baseline and follow-up data were expressed as mean (standard deviation) or median (interquartile range) for continuous variables and as a number (percentage) for categorical or binary variables. All continuous data were tested for a normal distribution using Kolmogorov-Smirnov nonparametric tests. Sphericity was tested using Mauchly’s test. Serial echocardiographic changes were assessed using a repeated measures analysis of variance. Post hoc analyses were performed using Bonferroni’s method. The cumulative event-free survival curve was determined according to the Kaplan-Meier method. Two-sided *p* values < 0.05 were considered statistically significant.

## Results

### General outcomes

The median postoperative follow-up duration was 54.1months. The overall mortality rate was 3.7 % (1 of 27): a 72-year-old woman died from septic shock caused by cholangitis, pancreatitis, and pneumonia, and had a pre-existing diagnosis of stomach cancer—despite good immediate-postoperative cardiac function. None of the patients experienced valve-related complications such as thromboembolism bleeding events associated anticoagulation. During the follow-up, there was no recurrence of endocarditis and no reoperation. The 5 year survival rate was 96.3 %.

### Immediate-postoperative echocardiographic results

Comprehensive transthoracic echocardiography was performed before patient discharge, the mean postoperative period being 7.4 ± 2.4 days. Immediately postoperatively, all patients showed mitral regurgitation less than grade 1. The LV end-diastolic dimension (EDD) and LV end-systolic dimension (ESD) decreased from 57.2 ± 6.3 to 45.4 ± 6.2 mm (*p* < 0.001) and from 35.7 ± 6.7 to 32.5 ± 6.3 mm (*p* = 0.046),respectively. But the LV EF decreased from 64.4 ± 9.6 % to 54.5 ± 9.8 % (*p* = 0.002). The LA dimension and estimated pulmonary artery systolic pressure (PASP) also significantly decreased from 46.5 ± 5.9 to 34.4 ± 8.9 mm (*p* < 0.001) and from 37.1 ± 14.9 to 24.5 ± 6.9 mm Hg (*p* < 0.001), respectively.

### Last-follow-up echocardiographic results

**Fig. 1:**
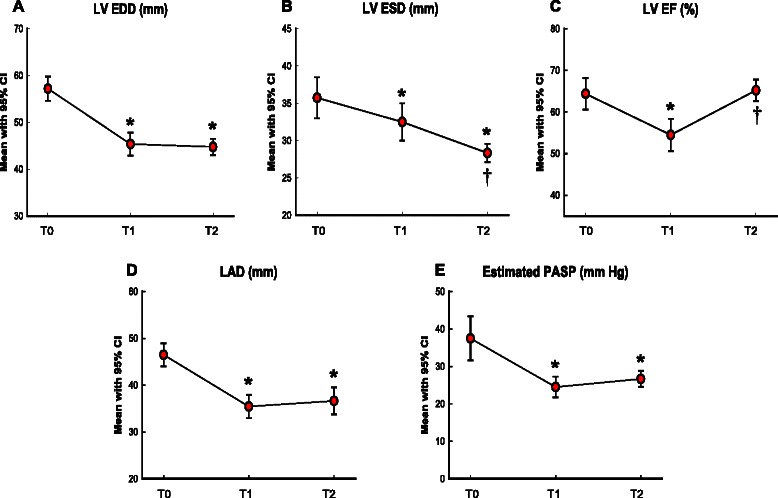
Serial changes of echocardiographic parameters: preoperative, median 7 days, and median 30 months after lifting mitral annuloplasty (T0, T1, and T2, respectively). **a** Left ventricular end-diastolic dimension (LV EDD, mm); **b** Left ventricular end-systolic dimension (LV ESD, mm); **c** Left ventricular ejection fraction (LV EF, %); **d** Left atrial dimension (LAD, mm); **e** Estimated pulmonary arterial systolic pressure (PASP, mm Hg) from tricuspid regurgitation peak velocity with estimated right atrial pressure. CI, Confidence interval. *P < 0.05, T0 versus T1 and T2, †*P* < 0.05, T1 versus T2, by post hoc Bonferroni’s test

The mean last-follow-up echocardiography interval was 30 ± 22 months. All of the survivors (26 of total 27) in our series had trivial or mild mitral regurgitation at their last follow-up visit. Follow-up LV reversal remodeling measured by the change in LV EDD, ESD, and EF was observed after surgery. The LV EDD and ESD decreased significantly compared with preoperative findings, from 57.2 ± 6.3 to 44.8 ± 4.1 mm (*p* < 0.001) and 35.7 ± 6.7 mm to 28.3 ± 2.9 mm (*p* < 0.001). The decreased LV EF in the immediate postoperative period improved from 54.5 ± 9.8 % to 65.2 ± 6.1 % (Fig. 1). The LA dimension and estimated PASP persistently decreased from 46.5 ± 5.9 to 35.3 ± 9.6 mm (*p* < 0.001) and 37.1 ± 14.9 to 26.9 ± 5.1 mmHg (*p* = 0.012). The mean mitral valve area measured by two-dimensional area was 2.27 ± 0.48 cm^2^ at the last follow-up visit. The echocardiographic findings are summarized in Table 3 and Fig. 1.

**Table 3:** Post-operative echocardiographic findings

	Pre-operative	Post-operative			
		Immediately after	*P* value*	Last follow-up	*P* value*
Follow-up duration		7.4 ± 2.4 days		30 ± 22 months	
MR ERO (cm^2^)	0.59 ± 0.30				
MR grade	3.68 ± 0.50	0.1 ± 0.2	<0.001	0.37 ± 0.22	<0.001
0 ~ I	0	27		26	
II	1	0		0	
III	4	0		0	
IV	22	0		0	
MVA, 2D (cm^2^)				2.27 ± 0.48	
LV EF (%)	64.4 ± 9.6	54.5 ± 9.8*	0.002	65.2 ± 6.1**	1.000
LV EDD (mm)	57.2 ± 6.3	45.4 ± 6.2*	<0.001	44.8 ± 4.1*	<0.001
LV ESD (mm)	35.7 ± 6.7	32.5 ± 6.3*	0.046	28.3 ± 2.9***	<0.001
LAD (mm)	46.5 ± 6.0	35.4 ± 6.3*	<0.001	36.6 ± 6.8*	<0.001
PASP (mmHg)	37.5 ± 14.2	24.5 ± 6.6*	<0.001	26.7 ± 5.0*	0.012

*MR* mitral regurgitation, *ERO* effective regurgitant orifice, *MVA* mitral valve area, *2D* two-dimensional, *LV* left ventricular, *EF* ejection fraction, *EDD* end-diastolic dimension, *ESD* end-systolic dimension, *LAD* left atrial dimension, *PASP* pulmonary artery systolic pressure estimated by tricuspid regurgitation peak velocity with right atrial pressure

*compared with preoperative

**P < 0.01, immediate after versus last follow-up

## Discussion

Mitral valve repair is a standard treatment for noninfected valvular disease. One of the advantages of mitral valve repair over mitral valve replacement is the preservation of LV function. Kouris and associates reported that mitral valve repair in patients with mitral regurgitation achieved better preservation of LV systolic indices than mitral valve replacement, probably due to preservation of the subvalvular apparatus and LV geometry [16], and several studies have suggested better survival and freedom from anticoagulant related hemorrhage, thromboembolism, and endocarditis [17, 18]. For these reasons, mitral valve repair is preferred to valve replacement in patients with degenerative mitral valve disease. Already the use of mitral valve repair has exceeded the use of replacement. Gammie et al. demonstrated that the rate of mitral valve repair versus replacement increased from 51 to 69 % and that operative risks of mitral valve repair were significantly lower than those for mitral valve replacement [19]. Barnett et al. reported that the surgical treatment for mitral disease has transitioned to primarily one of repair, not replacement [20]. But valvular replacement has been performed only infrequently for patients with mitral valve endocarditis because of the fragile valvular apparatus and concerns about recurrent IE and mitral valve plasty durability. Gammie et al. reported a 20 and 48 % feasibility rate of mitral valve repair in active and healed endocarditis respectively [8].

The most recent reports suggest acceptable short- and long-term outcomes. Ruttmann et al. demonstrated that event-free survival at 1 year was 88.2 and 80.4 % at 5 years in the repair group [4]. Miura et al. reported that 4-year survival was 88.2 % and the 4-year reoperation-free rate was 92.4 % in the repair group [21]. Shimokawa et al. reported that freedom from moderate or severe MR was 91.9 % ± 1.5 % and 83.3 % ± 2.3 % at 10 years [5]. Zegdi et al. reported that the 10-year survival rate and freedom from mitral valve reoperation were 80 and 91 %, respectively [7]. In our series, the overall mortality rate was 3.7 % (1 of 27) and the 5 year survival rate was 96.3 %.

In active endocarditis, the feasibility of valve repair depends on the extent of the pathological lesion [22]. In published studies, the feasibility of repairing infected mitral valves for acute endocarditis has been demonstrated to vary from 33 to 78 % [1]. Iung et al. reported that MV repair was feasible in 81 % of patients; 19 % of patients underwent MVR because of the extent of tissue destruction [23]. In our study, among the consecutive patients who underwent open heart surgery due to single mitral valve IE, only two patients (2 of 29) had replacements, both of them re-doing prior mitral valve operations. The extent of pathological lesions seemed not to restrict the choice for repair over replacement—most (15 of 27) involved both anterior and posterior leaflets, with the clinical and echocardiographic findings showing favorable outcomes.

Although an important surgical rule when dealing with valve surgery due to IE is to avoid the use of prosthetic material as often as possible, in our case, using the LMA strip with or without bovine pericardium to reconstruct the mitral valve defect, there was no recurrent endocarditis during the median follow-up of 54 months. The reported recurrence rates of endocarditis in patients undergoing mitral valve repair for active endocarditis were as low as 1 in 37 patients [7]. In our study, we do not confirm that the mitral strip was tolerant of infection, but we believe that our novel technique of LMA with a strip, with or without PLA, helped to increase the reconstructibility in active endocarditis as to 27 of 29 (93 %), which overall helps reduce the recurrence of endocarditis in surgically treated active IE. Radical debridement of infective tissue followed by local antibiotic irrigation and fully maintained intravenous antibiotic therapy for 4–6 weeks are, we believe, important concomitants in preventing recurrence of IE [24].

In patients with rheumatic valve pathology such as shrunken or thickened leaflets, we sliced the leaflet to increase pliability and then augmented the posterior leaflet using bovine pericardium. This technique has been described by Chauvaud et al. [13]. We used a bovine pericardium because the use of this material has been shown to be more cost-effective although no data are available on the long-term results of the use of bovine pericardium—longer follow-up is essential.

The normal mitral annulus is not a two-dimensional flat and fixed structure but rather takes the three-dimensional form of a saddle [25]. The saddle shape is accentuated from late diastole to early systole in the normal annulus but this change is blunted in mitral valve disease [26]. This shape has been shown to reduce stress on the mitral leaflet, an important determinant of durability after mitral valve repair [27]. Our lifting annuloplasty was based on this concept. Anatomically, the anterior mitral annulus attaches to the fibrous skeleton at the base of the heart and is more rigid; the posterior annulus is thinner and more susceptible to dilatation [28]. With LMA, annular sutures go through the LA wall above the posterior mitral annulus, then slightly go through the annulus and out through LA wall. This annular suture technique has the effect of lifting the posterior annulus to the LA side and increasing the annulus three-dimensional curvature. In this study, in patients with active IE, repair of mitral valve using LMA with or without PLA reveals good outcomes with an acceptable coaptation of both mitral leaflets.

A decrease in atrial and ventricular size, called reverse remodeling, has been observed after mitral valve procedures. In our study, the immediate postoperative LV EF was decreased but recovered after follow-up. This phenomenon may be because the LV EDD is reduced more quickly than the LV ESD, resulting in a decreased LV EF; however, the negative remodeling of the LV ESD restored the LV EF (Fig. 1). These findings mean that reverse remodeling may need significant time.

### Limitations

This is a single-center experience with a relatively small number of patents. Our study excluded patients having mitral valve replacement in order to focus on the effects of our techniques on mitral valve repair with endocarditis per se, and comparison between mitral valve repair and replacement was limited due to only 2 patients undergoing replacement. Finally, we are aware that the lack of comparison with other repair techniques limits conclusions concerning the superiority of the LMA approach. However, repair techniques using bovine pericardium and LMA has enabled us to suggest that extensive IE is not a strong contraindication for mitral valve repair. Further studies incorporating a larger number of patients and comparable groups using other repair techniques are necessary to evaluate the relative advantages of our mitral valve repair techniques.

## Conclusion

This repair technique of LMA with or without PLA in patients with acute-phase IE achieved good structural and functional outcomes as well as an excellent 5 year survival rate. An individualized repair approach should be recommended in patients with acute-phase IE.

## Abbreviations

EDD: End-diastolic dimension
EF: Ejection fraction
ERO: Effective regurgitant orifice
ESD: End-systolic dimension
IE: Infective endocarditis
LA: Left atrial
LMA: Lifting mitral annuloplasty
LV: Left ventricular
PASP: Pulmonary artery systolic pressure
PLA: Posterior leaflet augmentation
